# Algorithms for target transformations of lattice basis vectors

**DOI:** 10.1107/S2053273320012668

**Published:** 2020-10-29

**Authors:** Semën Gorfman

**Affiliations:** a Department of Materials Science and Engineering, Tel Aviv University, Wolfson Building for Mechanical Engineering, Tel Aviv 6997801, Israel

**Keywords:** crystal lattice, transformations, lattice planes, zones

## Abstract

Simple algorithms are proposed for the transformation of lattice basis vectors to a specific target. In the first case, one of the new basis vectors is aligned to a predefined lattice direction, while in the second case, two of the new basis vectors are brought to a lattice plane with predefined Miller indices. The multi-dimensional generalization of the algorithm is available in the supporting materials. The algorithms are useful for such crystallographic operations as simulation of zone planes (*i.e.* geometry of electron diffraction patterns) or transformation of a unit cell for surface or cleavage energy calculations. The most general multi-dimensional version of the algorithm may be useful for the analysis of quasiperiodic crystals or as an alternative method of calculating Bézout coefficients. The algorithms are demonstrated both graphically and numerically.

## Abstract 2

Presented here are algorithms for the transformation of lattice basis vectors to a specific target. The algorithms are useful for crystallographic operations in direct and reciprocal spaces alike. The algorithms are demonstrated graphically and numerically.

## 1. Introduction   

The transformation of lattice basis vectors is a key mathematical operation in crystallography. It is expressed using a transformation matrix [*S*] (**A**_*i*_ = **a**_*j*_ *S*_*ji*_) connecting old (**a**_1_, **a**_2_, **a**_3_), and new (**A**_1_, **A**_2_, **A**_3_) lattice basis vectors. If det[*S*] = ±1 and 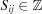, then **A**_*i*_ and **a**_*i*_ are the bases of the same lattice/form the unit cells of the same crystal structure. Such unit-cell transformations are useful for analysing the structures of polymorphs (Müller, 2013 ▸; de la Flor *et al.*, 2016 ▸), twin laws (Nespolo & Ferraris, 2006 ▸; Zhang *et al.*, 2010 ▸; Marzouki *et al.*, 2014 ▸), phase transitions (Howard & Stokes, 2005 ▸), tilting of structural polyhedra (Glazer, 1972 ▸, 1975 ▸) and nanoscale stacking order (Biermanns *et al.*, 2011 ▸). The ability to ‘view’ the same crystal structure using different unit-cell settings is crucial for a skilful crystallographer.

This article introduces new algorithms for the transformation of basis vectors for a specific target. The first version of the algorithm enables the transformation (**A**_*i*_ = **a**_*j*_ *S*_*ji*_, det[*S*] = ±1) such that **A**_3_ is parallel to a target lattice vector **T**. The second version results in **A**_1_, **A**_2_ parallel to a target lattice plane (*hkl*) and **A**_3_ connecting lattice points of two adjacent planes. In this way the algorithm suggests an alternative approach to calculate the Bézout coefficients (Bézout, 1779 ▸).

In contrast with other number-theoretical approaches [*e.g.* the extended Euclidean algorithm (Knuth, 1997 ▸)], the new algorithms are easily extendable to higher-dimensional lattices. In addition, two- and three-dimensional versions allow for clear visualization using lattice directions and their stereographic projections. The algorithms are useful for the simulation of electron diffraction patterns or for exploring the two-dimensional periodicity of crystal structures within a given lattice plane. It is necessary to follow the algorithms whenever the indices of a target direction or a plane are non-trivial. The output can be used in structure visualization programs [*e.g.* *VESTA* (Momma & Izumi, 2011 ▸)], for the structure utilities of the Bilbao Crystallographic Server (Aroyo, Perez-Mato *et al.*, 2006 ▸; Aroyo, Kirov *et al.*, 2006 ▸) and for the *ab initio* calculation of surface energy (Tran *et al.*, 2016 ▸; Kresse & Furthmüller, 1996 ▸; Schwarz *et al.*, 2002 ▸). The multi-dimensional version of the algorithm might be useful for the analysis of quasiperiodic materials. The algorithm is deposited as a MATLAB program.

## 2. Transformation to a target direction   

The list of notations and relevant crystallographic relations are available in Appendix *A* [App appa]. The names of the two-, three- and multi-dimensional algorithms are *PARA*, *TRIO* and *MULDIN*, respectively. *PARA* and *TRIO* are described here, while *MULDIN* is deposited in the supporting information.

### 2.1. *PARA*: two-dimensional lattice   

This algorithm transforms the basis vectors **a**_1_, **a**_2_ to **A**_1_, **A**_2_ such that **A**_2_ ∥ **T** = *x*_1_ **a**_1_ + *x*_2_ **a**_2_. Table S1 and Fig. S1 (in the supporting information) provide a step-by-step illustration of the algorithm for the example target vector **T** = 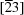.

**Iteration 0:** We transform the basis vectors **a**_*i*_ to 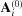 = ±**a**_*i*_ (‘−’ is taken if *x*_*i*_ is negative) and rearrange them so that det[*S* ^(0)^] = 1, where [*S* ^(0)^] is a 2×2 transformation matrix 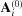 = 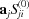 [Fig. S1(*a*)]. According to equation (19) in Appendix *A* [App appa] the new coordinates of **T** are [**X** ^(0)^] = [*S* ^(0)^]^−1^[**x**]. All the components of [**X** ^(0)^] are non-negative.

**Iteration *n*:** We replace one of the basis vectors by 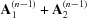. This creates two transformation variants [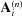 = 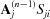],
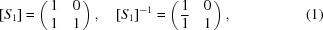


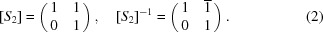
Because det[*S*] = 1, 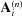 form the basis of the same lattice for both variants. The coordinates of **T** transform as 
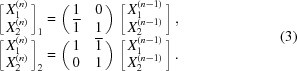
The exit condition of the algorithm is that one of 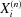 is zero. This happens if 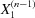 = 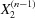. If valid, the exit condition holds for both transformation variants. We can choose variant (2) to ensure that 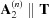 . The final transformation **a**_*i*_ → **A**_*i*_ is given by the matrix product, 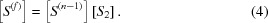
If the exit condition is not fulfilled, we select the variant *m* yielding all positive 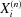: 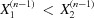 and 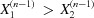 force the choice of *m* = 1 [Fig. S1(*c*)] and *m* = 2 [Fig. S1(*b*)], respectively. The transformation 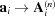 is 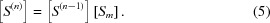
The algorithm continues to the next iteration until the exit condition is reached [Fig. S1(*d*)].

### 2.2. *TRIO*: three-dimensional lattice   

**Figure 1:**
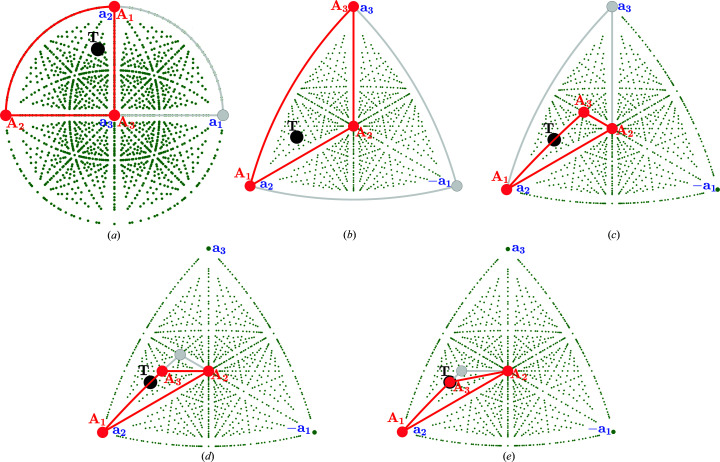
The transformations of the basis vectors given in the rows of Table 1 ▸: panel (*a*) corresponds to the first row in Table 1 ▸ with *n* = 0, panel (*b*) to the second row with *n* = 1 *etc*. The directions are drawn on the stereographic projection, which includes the poles of a cubic crystal lattice with a maximum index of 10. Panel (*a*) uses the stereographic projection along [001], and panels (*b*) to (*d*) use the stereographic projection along 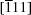.

This algorithm transforms the basis vectors of a three-dimensional lattice **a**_*i*_ → **A**_*i*_ so that **A**_3_ ∥ **T** = *x*_*i*_ **a**_*i*_. Table 1 ▸ and Fig. 1 ▸ support the explanations.

**Iteration 0:** We transform the basis vectors 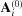 = ±**a**_*i*_ as in *PARA*, followed by their permutations ensuring the ‘right-handedness’ of 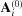 {det[*S* ^(0)^] = 1}, where [*S* ^(0)^] is a 3×3 transformation matrix between **a**_*i*_ and 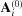 [Fig. 1 ▸(*a*)]. The new non-negative coordinates of **T** become [**X** ^(0)^] = [*S* ^(0)^]^−1^[**x**].

**Iteration n:** We replace one of the basis vectors by 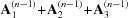, creating three transformation variants [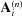 = 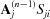]: 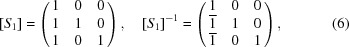


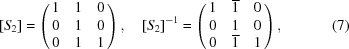


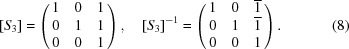
Because det[*S*] = 1, 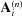 and **a**_*i*_ build the same lattice for all three variants. The new coordinates of **T** are 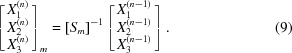
The exit condition (two of the new **T** coordinates are zero) is fulfilled if 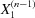 = 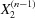 = 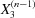. If valid, it holds for all three variants but choosing [*S*_3_] ensures 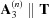. The transformation **a**_*i*_ → **A**_*i*_ is [*S* ^(*f*)^] = [*S* ^(*n*−1)^][*S*_3_]. Otherwise, we select the variant *m*, which gives non-negative 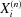. According to equations (6)–(9) [Disp-formula fd8] [Disp-formula fd9], *m* is defined such that 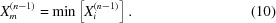
If equation (10) is fulfilled only for one *m* then none of 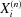 is zero [Fig. 1 ▸(*b*), *m* = 2]. The transformation 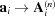 is 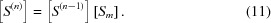
This moves the algorithm to the iteration *n* + 1.

However, equation (10) might be valid for two variants if *e.g.* 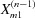 = 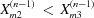. Then either [*S*_*m*1_] or [*S*_*m*2_] could be selected for the next iteration, which yields 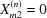 or 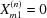, respectively. The transformation 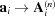 is described by equation (11) with *m* = max(*m*1, *m*2), which ensures 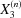 ≠ 0 [*m* = 3, 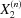 = 0 in Fig. 1 ▸(*c*)]. The algorithm will be completed by *PARA* with respect to the vectors **a**_1_ = 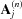 and **a**_2_ = 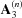 such that 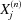 ≠ 0 [Figs. 1 ▸(*d*) and 1 ▸(*e*)].

Table 1 ▸ and Fig. 1 ▸ show that the example of the *TRIO* algorithm where the target vector is **T** = 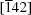 results in
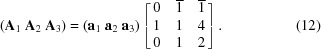



## 3. Transformation of basis vectors to a target lattice plane   

We will show how *TRIO* helps transform the basis vectors **a**_*i*_ → **A**_*i*_ so that **A**_1_, **A**_2_ are parallel to the reticular (lattice) planes with Miller indices (*hkl*). Such planes are perpendicular to a reciprocal-lattice vector T* = 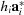, and the inter-planar distance is the inverse length of T* [see *e.g.* De Graef & McHenry (2012 ▸) and Giacovazzo (1992 ▸)]. We apply *TRIO* to the reciprocal basis vectors 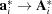 with the target vector 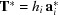:
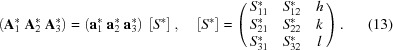
We can calculate the equivalent matrix of transformation of the direct-lattice vectors [equation (21) in Appendix *A* [App appa]], 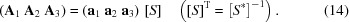
This will result in **A**_1_, **A**_2_ ⊥ T*. Because **A**_*i*_ form the basis of the same lattice, **A**_1_, **A**_2_ are the basis of the two-dimensional lattice in the (*hkl*) planes and **A**_3_ connects two adjacent planes. This algorithm applies for two-dimensional (Table S2/Fig. S2) and multi-dimensional cases alike. The three-dimensional case is illustrated in the key application below and in Table S3/Fig. S3. The multi-dimensional version (supporting information) may be useful for the analysis of quasicrystals where projecting the multi-dimensional lattice onto one of its three-dimensional sub-lattices is required.

Note that the coordinates of **A**_3_ (or vector **A**_*N*_ for the multi-dimensional case) are known as Bézout coefficients (Bézout, 1779 ▸). Therefore, the algorithm may be useful as an alternative method for finding such coefficients for any number of dimensions.

## 4. Simulation of the geometry of zone planes perpendicular to the target zone axis   

The ‘zone axis’ is the direction parallel to a lattice vector **T** = *u*_*i*_ **a**_*i*_ (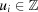). The reciprocity of direct and reciprocal lattices means that **T** is normal to the reciprocal-lattice planes with ‘Miller’ indices *u*_*i*_. Zones appear *e.g.* in electron diffraction as two-dimensional sections of reciprocal space by a nearly flat Ewald sphere (Vainshtein, 2013 ▸) or in Laue diffraction patterns (Helliwell *et al.*, 1989 ▸; Ren *et al.*, 1999 ▸; Send *et al.*, 2009 ▸) as visually striking second-order curves – ellipses, hyperbolas and parabolas.

**Figure 2:**
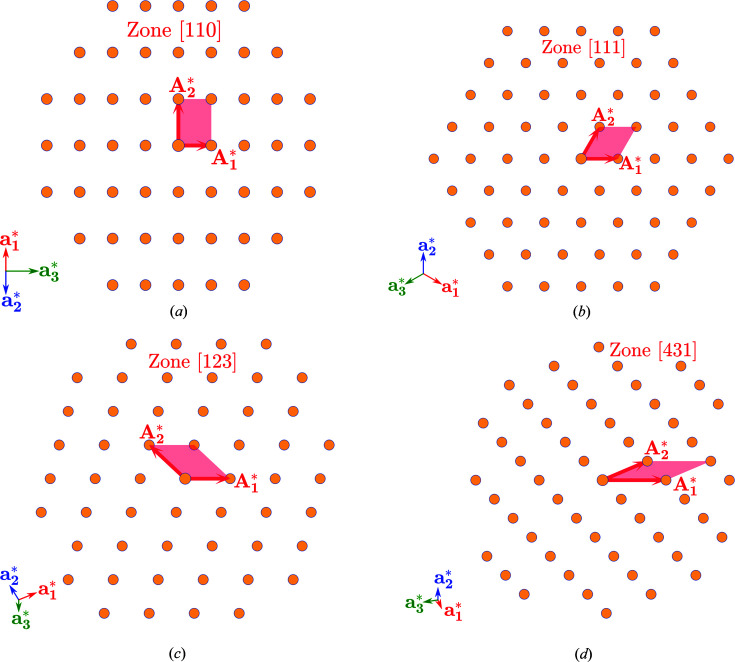
Illustrations of the new basis vectors for a cubic lattice and pre-selected zone axes, corresponding to the rows in Table 2 ▸: panel (*a*) corresponds to the first row in Table 2 ▸ with zone axis [110], panel (*b*) to the second row with zone axis [111] *etc*. The projection of the basis vectors 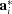 is shown in the bottom left-hand corner of each panel.

Using **T** as a target of *TRIO*, we obtain the transformation matrix [*S*] for the direct-lattice vectors with **A**_3_ ∥ **T**. The corresponding reciprocal-lattice vectors 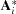 = 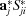 are transformed by [S*]^T^ = [*S*]^−1^, with 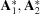 parallel to the zone plane. The two-dimensional lattice parameters of a zone are obtained from the components of the reciprocal metric tensor and equation (22):
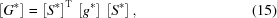
followed by
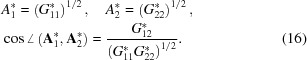
Table 2 ▸ and Fig. 2 ▸ demonstrate four ‘zones’ of a cubic lattice (**a**_*i*_ **a**_*j*_ = *a* ^2^δ_*ij*_) with *a* = 4 Å.

Section S5 in the supporting information shows another application of the algorithm for the transformation of a unit cell (the LiNbO_3_ crystal structure is considered). In particular, it demonstrates the extension of the algorithm for the case of a non-primitive conventional unit cell.

## 5. On the length of the vectors *A*_*i*_   

The algorithms introduced here help in reaching one of infinitely many transformations to the specific target. However, the course of the algorithms does not depend on the matrix of dot products [*G*] or lattice parameters. Under these conditions, the transformed lattice parameters remain undefined and it is therefore impossible to choose one variant of the transformation over another. If [*G*] is known, the existing lattice reduction algorithms [*e.g.* Niggli (1928 ▸)] can be applied to transform the subset of **A**_*i*_ (*e.g.* **A**_1_ and **A**_2_) without changing the target. For example, it is possible to apply the Minkowski algorithm to reduce the lengths of vectors **A**_1_ and **A**_2_ [*e.g.* Rote (1997 ▸) and Helfrich (1985 ▸)].

## 6. Related literature   

The following references are cited in the supporting information: Abrahams *et al.* (1966 ▸), Ong *et al.* (2013 ▸), Weis & Gaylord (1985 ▸).

## 7. Conclusions   

We have presented algorithms for the transformation of lattice basis vectors, so that one of the vectors is parallel to a target direction **T**, or alternatively two of the vectors are parallel to the target lattice planes (*hkl*). Such transformations are useful for *e.g.* the simulation of electron diffraction (presented here) and the transformation of crystal structures for exposing certain lattice planes (supporting information). We generalize the algorithm to the multi-dimensional case (*MULDIN* algorithm, supporting information), which may be useful for the analysis of quasiperiodic crystals or as an alternative approach for finding multi-dimensional Bézout coefficients.

## Supplementary Material

MATLAB script realizing the algorithm in the general multi-dimensional form. The number of space dimensions is determined by the size of the input vector. DOI: 10.1107/S2053273320012668/ae5090sup1.txt

Additional tables and figures. DOI: 10.1107/S2053273320012668/ae5090sup2.pdf

## Figures and Tables

**Table 1:** Information for each *TRIO* iteration for the transformation to the target vector **T** = 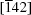 The table is organized in the same way as Table S1. Iterations 3 and 4 in this example follow the *PARA* algorithm. For the first row [*X* ^(−1)^] = [*x*].

*n*	[**X** ^(*n*−1)^]	[*S*_*m*_]	[*S* ^(*n*)^]	[**X** ^(*n*)^]
0	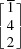		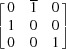	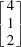
1	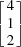	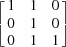	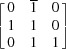	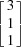
2	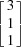	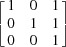	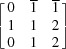	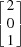
3	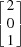	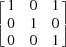	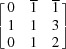	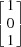
4	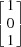	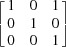	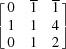	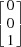

**Table 2:** The transformations of the reciprocal-lattice basis vectors of a cubic lattice (*a* = 4 Å) to pre-selected zones The last column shows the corresponding two-dimensional lattice parameters.

Zone axis	[S*]	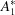 , 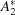 , 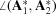
[110]	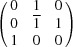	0.25 Å^−1^, 0.36 Å^−1^, 90°
[111]	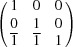	0.35 Å^−1^, 0.35 Å^−1^, 60°
[123]	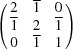	0.56 Å^−1^, 0.61 Å^−1^, 137°
[431]	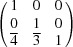	1.03 Å^−1^, 0.79 Å^−1^, 23°

## Appendix A Symbols, notation and important crystallographic relationships

The indices *i*, *j* run from 1 to *N* (*N* is the number of space dimensions). The Einstein summation rule applies to these indices but is abandoned for the index in brackets (*m*).

**a**_*i*_ are linearly independent basis vectors of an *N*-dimensional crystal lattice. **A**_*i*_ are transformed basis vectors of the same crystal lattice. We assume that both sets determine primitive unit cells, otherwise the transformation to such must be performed.

are the reciprocal basis vectors, such that their dot products with **a**_*i*_ are  = δ_*ij*_ (here δ_*ij*_ is the Kronecker delta).  are the reciprocals of the transformed basis vectors such that  = δ_*ij*_.

[*g*] and [*g**] are the components of the direct and reciprocal metric tensors: *g*_*ij*_ = **a**_*i*_ · **a**_*j*_ and  = . [*G*] and [*G**] are the analogous components for the transformed basis vectors: *G*_*ij*_ = **A**_*i*_ · **A**_*j*_ and  = .

*x*_*i*_ are the direct lattice coordinates of a vector **T** = *x*_*i*_ **a**_*i*_. *X*_*i*_ are the coordinates of the same vector relative to the transformed basis vectors: **T** = *X*_*i*_ **A**_*i*_.

*h*_*i*_ (*hk* for two- and *hkl* for three-dimensional cases) are the reciprocal-lattice coordinates of a vector **T*** = . *H*_*i*_ are the transformed coordinates of the same vector: **T*** = .

[*S*] is the *N*×*N* matrix of the **a**_*i*_ → **A**_*i*_ transformation. The columns of [*S*] are the coordinates of **A**_*i*_ relative to **a**_*i*_, so that **A**_*i*_ = **a**_*j*_ *S*_*ji*_. For the three-dimensional case, the formal matrix equation applies:

**A**_*i*_ form a primitive unit cell of the same lattice if  and det[*S*] = ±1. (det[*S*] = −1 if the transformation changes the handedness of the coordinate system.)

[*S**] is the *N*×*N* matrix of transformation for the reciprocal-lattice vectors:

[**x**], [**X**] are the *N*×1 columns of the numbers *x*_*i*_, *X*_*i*_, respectively. [**h**], [**H**] are the 1×*N* rows of the reciprocal-lattice coordinates *h*_*i*_, *H*_*i*_.

The following transformations are used in this paper (De Graef & McHenry, 2012 ▸; Hahn, 2005 ▸; Giacovazzo, 1992 ▸).

The transformation of the direct- and reciprocal-lattice coordinates of a vector:

The relationship between [*S*] and [*S**]:

The transformation of the matrix of the dot product:

If the transformations  and  are defined by the matrices [*S*_1_] and [*S*_2_], then the combined transformation  is defined as a matrix product:
