# Supplementary material for: Algorithms for target transformations of lattice basis vectors
Source: Acta Crystallogr A Found Adv. 2020 Oct 29;76(Pt 6):713–8. doi: 10.1107/S2053273320012668 (PMC7598097; doi:10.1107/S2053273320012668)
Supplement: Supplementary file 2 [file a-76-00713-sup2.pdf]

FOUNDATIONS
ADVANCES

Volume 76 (2020)

Supporting information for article:

Algorithms for target transformations of lattice basis vectors

Semën Gorfman

S1. Illustration of the convergence of the PARA algorithm for the example target vector $\boldsymbol{T} = [\bar{2}\ 3]$.

Table S1 The information about each PARA iteration converging to the example target vector $\boldsymbol{T} = [\bar{2}\ 3]$. Column 2 shows the coordinates of this vector from the previous iteration, columns 3 and 4 show the matrices $[S_m]$ and $[S^{(n)}]$ of transformations $\boldsymbol{A}_i^{(n-1)} \to \boldsymbol{A}_i^{(n)}$ and $\boldsymbol{a}_i \to \boldsymbol{A}_i^{(n)}$ ($[S^{(n)}] = [S^{(n-1)}]\,[S_m]$). The last column shows the coordinates of the target vector at the end of each iteration. For the first raw $[\boldsymbol{X}^{(-1)}] = [\boldsymbol{x}]$

Figure S1 The graphical illustration of the transformation of basis vectors in Table S1. The figures include a fragment of a crystal lattice and the target vector $\boldsymbol{T} = [\bar{2}\ 3]$. Figure S1(a) includes initial basis vectors $\boldsymbol{a}_1$ and $\boldsymbol{a}_2$, Figure S1(b-d) includes the basis vectors $\boldsymbol{A}_i = \boldsymbol{A}_i^{(n)}$ at the end of each iteration. The values of lattice parameters are irrelevant for the course of this algorithm and thus arbitrarily chosen.

n	$[\boldsymbol{X}^{(n-1)}]$	$[S_m]$	$[S^{(n)}]$	$[\boldsymbol{X}^{(n)}]$
0	$\begin{bmatrix}\bar{2}\\3\end{bmatrix}$		$\begin{pmatrix}0 & \bar{1}\\1 & 0\end{pmatrix}$	$\begin{bmatrix}3\\2\end{bmatrix}$
1	$\begin{bmatrix}3\\2\end{bmatrix}$	$\begin{pmatrix}1 & 1\\0 & 1\end{pmatrix}$	$\begin{pmatrix}0 & \bar{1}\\1 & 1\end{pmatrix}$	$\begin{bmatrix}1\\2\end{bmatrix}$

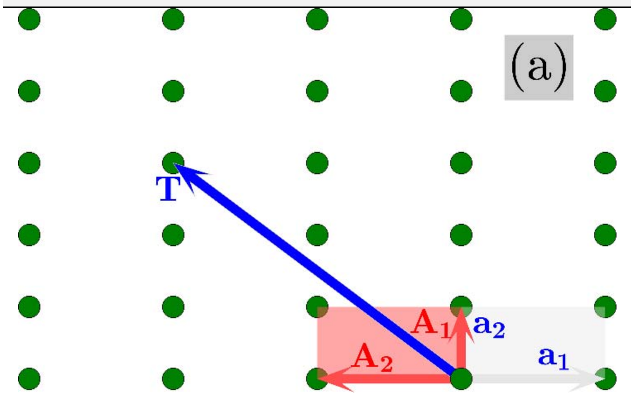

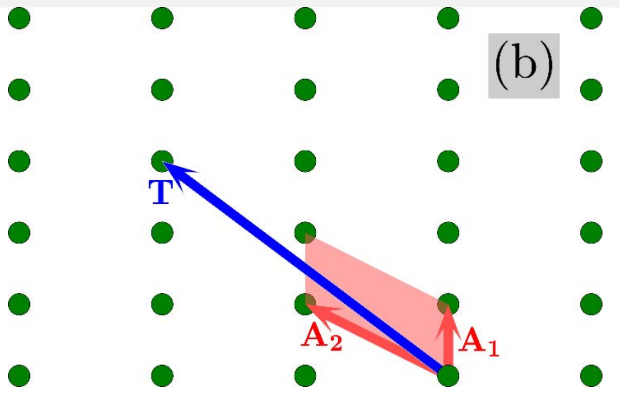

2	$\begin{bmatrix}1\\2\end{bmatrix}$	$\begin{pmatrix}1 & 0\\1 & 1\end{pmatrix}$	$\begin{pmatrix}\bar{1} & \bar{1}\\2 & 1\end{pmatrix}$	$\begin{bmatrix}1\\1\end{bmatrix}$	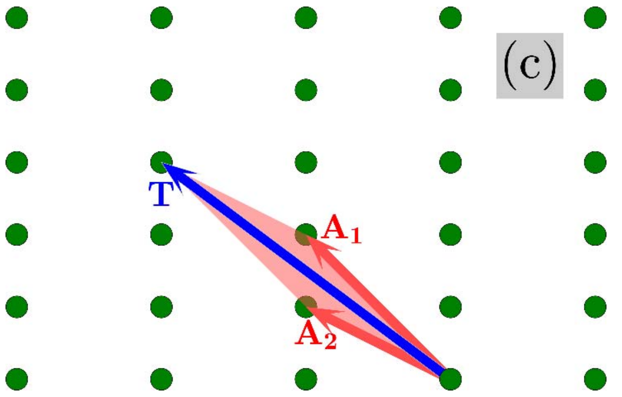
3	$\begin{bmatrix}1\\1\end{bmatrix}$	$\begin{pmatrix}1 & 1\\0 & 1\end{pmatrix}$	$\begin{pmatrix}\bar{1} & \bar{2}\\2 & 3\end{pmatrix}$	$\begin{bmatrix}0\\1\end{bmatrix}$	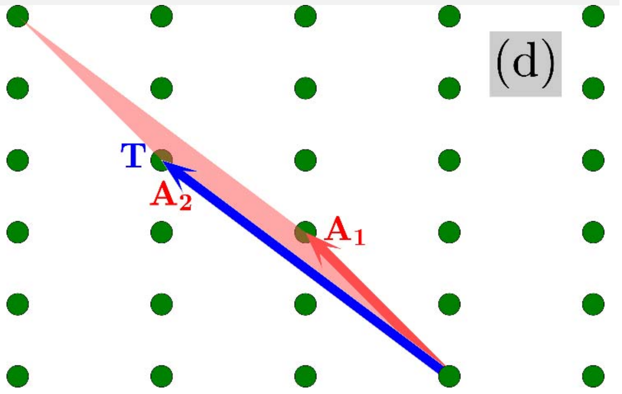

S2. MULDIN: Generalization of PARA / TRIO algorithm for *N*-dimensional crystal lattice

MULDIN is the general version of PARA and TRIO. It transforms the basis vectors of N-dimensional lattice $\boldsymbol{a}_i$ to $\boldsymbol{A}_i$ in such a way that $\boldsymbol{A}_N \parallel \boldsymbol{T} = x_i\boldsymbol{a}_i$ and the new vectors build the same lattice, i.e. determinant of the $N \times N$ transformation matrix is 1. We assume that at least two x_i is non-zero, otherwise the solution to the problem is achieved by cyclic permutation of the basis vectors such that $X_N \neq 0$.

Iteration 0:

The transformation $\boldsymbol{A}_i^{(0)} = \pm\boldsymbol{a}_i$ as in PARA is performed. This is followed by the permutation of the transformation matrix columns ensuring that $\det[S^{(0)}] = 1$ and that $X_N^{(0)} \neq 0$ (here $[\boldsymbol{X}^{(0)}] = [S^{(0)}]^{-1}[\boldsymbol{x}]$). All the elements of $[\boldsymbol{X}^{(0)}]$ are non-negative.

Iteration n:

We replace one of the basis vectors by $\sum c_i \boldsymbol{A}_i^{(n-1)}$ where $c_i = 0$ if $X_i^{(n-1)} = 0$ or $c_i = 1$ otherwise. This sum includes only the terms corresponding to non-zero coordinate of the target vector. Assuming that there are M of them, we can get M variants of the transformation matrix with:

$$S_{ij(m)} = \delta_{ij} + c_i\delta_{j(m)} - \delta_{i(m)}\delta_{j(m)} \tag{S2.1}$$

Here m are such that $c_m = 1$. The first term in the equation (S2.1) describes the unitary matrix. Adding the second and the third terms replace its column m by the column vector $[c]$. The determinant of such transformation matrix is equal to 1, therefore the transformed vectors build the same N-dimensional lattice. The components of the inverse matrix are:

$$S_{ij(m)}^{-1} = \delta_{ij} - c_i\delta_{j(m)} + \delta_{i(m)}\delta_{j(m)} \tag{S2.2}$$

The coordinates of the target vector are transformed according to the equation (A3):

$$X_{i(m)}^{(n)} = \begin{cases} X_i^{(n-1)} & i = m \; or \; c_i = 0 \\ X_i^{(n-1)} - X_m^{(n-1)} & \text{otherwise} \end{cases} \tag{S2.3}$$

The exit condition of the algorithm is that $(N-1)$ new coordinates of the target vector are zero. According to (S2.3) this happens if, and only if, all non-zero $X_i^{(n-1)}$ are equal to each other (X). If valid, the exit condition is fulfilled for all variants and $\boldsymbol{T} = X\boldsymbol{A}_m^{(n)}$. We can use the last variant, $m = N$, for the final transformation $\boldsymbol{a}_i \rightarrow \boldsymbol{A}_i$ is given by the matrix

$$\left[S^{(f)}\right] = [S^{(n-1)}]\,[S_N] \tag{S2.4}$$

Otherwise we choose such transformation variant (S2.1) that all $X_i^{(n)}$ in (S2.3) are non-negative. Such variant can be easily found by sorting the non-zero $X_i^{(n-1)}$ in the ascending order: $X_{m_1}^{(n-1)} = X_{m_2}^{(n-1)} \ldots = X_{m_K}^{(n-1)} < X_{m_K+1}^{(n-1)} \leq \cdots \leq X_{mM}^{(n-1)}$. As follows from (S2.3), any variant $m_1, m_2, \ldots, m_K$ corresponds to non-negative $X_{i(m)}^{(n)}$. However, some of the new coordinates are zero: if e.g. $m = m_1$ then $X_{m_2}^{(n)} \ldots = X_{m_K}^{(n)} = 0$. We can choose any of these m above, but ensuring that $X_N^{(n)} \neq 0$. For the algorithmic purposes we will fix m:

$$m = \max(m_1, m_2, \ldots, m_K) \tag{S2.5}$$

The coordinates of the new basis vectors $\boldsymbol{A}_i^{(n)}$, relative to the original basis vectors $\boldsymbol{a}_i$ are given by the matrix product:

$$\left[S^{(n)}\right] = [S^{(n-1)}]\,[S_m] \tag{S2.6}$$

This closes the iteration n and moves the algorithm to the iteration number $n + 1$.

S3. The examples and the illustrations of the transformation of two-dimensional basis to the target lattice rows.

The transformation of the vectors $\boldsymbol{a}_1, \boldsymbol{a}_2 \rightarrow \boldsymbol{A}_1, \boldsymbol{A}_2$ is performed in such a way that $\boldsymbol{A}_1$ is parallel to the target (hk) rows. The image illustrates the unit cell before and after the transformation with three adjacent lattice rows drawn.

Table S2 Examples of the target transformation of basis vectors to the preselected lattice rows with Miller indices (hk) (the column 1). Columns 2 and 3 show the matrices of transformation of reciprocal ($\boldsymbol{a}_i^* \rightarrow \boldsymbol{A}_i^*, \boldsymbol{A}_2^* \parallel \boldsymbol{T}^*$) and direct basis vectors ($\boldsymbol{a}_i \rightarrow \boldsymbol{A}_i, \boldsymbol{A}_1 \parallel (hk)$).

(hk)	$[S^*]$	$[S] = [[S^*]^{-1}]^T$
(11)	$\begin{pmatrix}1 & 1\\ 0 & 1\end{pmatrix}$	$\begin{pmatrix}1 & 0\\ \bar{1} & 1\end{pmatrix}$

Figure S2 Illustration of the transformation of the basis vectors to the target lattice rows (hk). The figures include original $\boldsymbol{a}_i$ and transformed $\boldsymbol{A}_i$ basis vectors along with three adjacent rows drawn. Note, that transformed Miller indices (HK) are always equal to (01). As in Figure S1, the values of lattice parameters are irrelevant for the course of this algorithm and thus arbitrarily chosen.

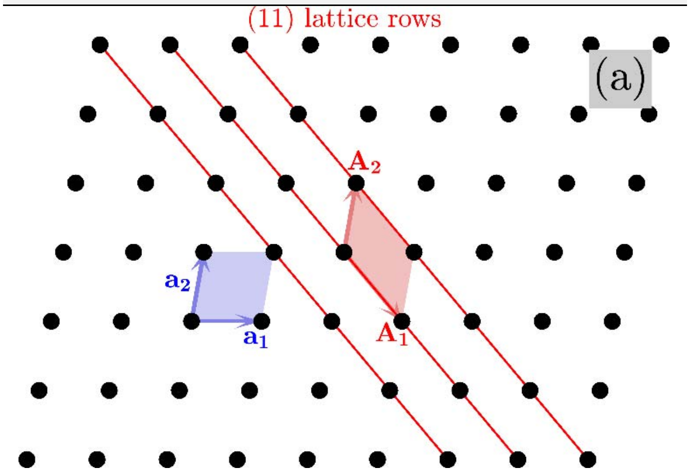

$(1\bar{2})$	$\begin{pmatrix} 0 & 1 \\ \bar{1} & \bar{2} \end{pmatrix}$	$\begin{pmatrix} \bar{2} & 1 \\ \bar{1} & 0 \end{pmatrix}$	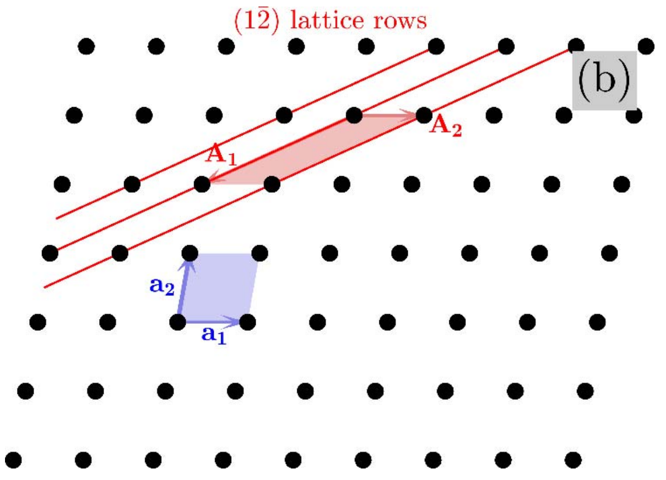
$(\bar{2}3)$	$\begin{pmatrix} \bar{1} & \bar{2} \\ 2 & 3 \end{pmatrix}$	$\begin{pmatrix} 3 & \bar{2} \\ 2 & \bar{1} \end{pmatrix}$	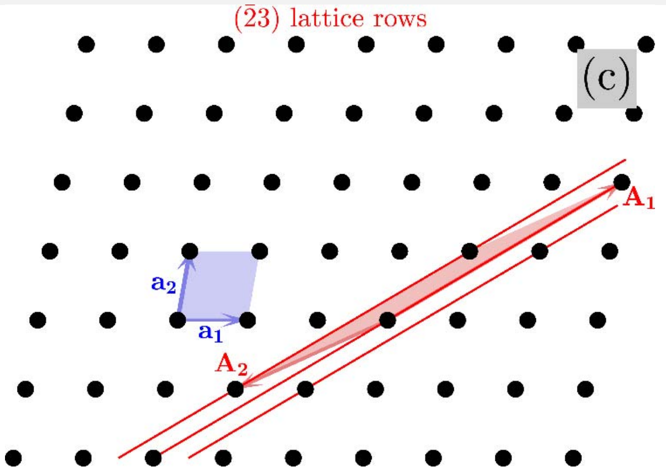
(31)	$\begin{pmatrix} 1 & 3 \\ 0 & 1 \end{pmatrix}$	$\begin{pmatrix} 1 & 0 \\ \bar{3} & 1 \end{pmatrix}$	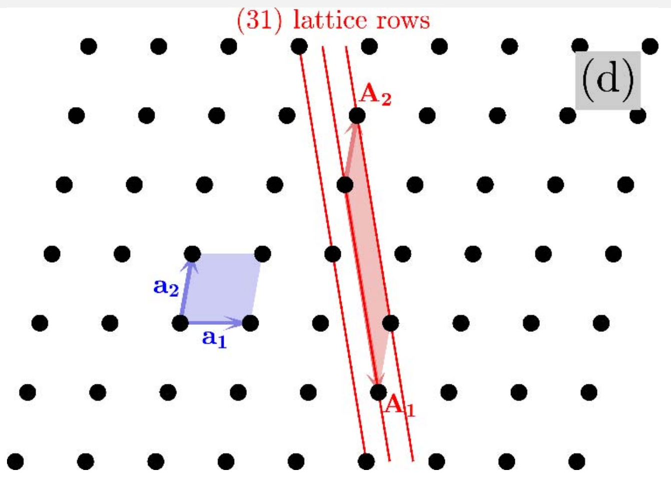

S4. The examples and illustration of the transformation of three-dimensional basis to the target lattice planes.

Table S3 and Figure S3 show the examples of transformation of 3D basis vectors $\boldsymbol{a}_1, \boldsymbol{a}_2, \boldsymbol{a}_3 \rightarrow \boldsymbol{A}_1, \boldsymbol{A}_2, \boldsymbol{A}_3$ in such a way that $\boldsymbol{A}_1$ and $\boldsymbol{A}_2$ are parallel to the target (hkl) lattice plane. The images show the original and transformed unit cells by means of the stereographic projections along $\boldsymbol{T}^*$. The stereographic projection of a unit cell directions before and after the transformation are drawn.

Table S3 Examples of the target transformation of basis vectors to the preselected lattice planes with Miller indices (hkl). The table is organized as Table S2.

(hkl)	$[S^*]$	$[[S^*]^{-1}]^T$
(111)	$\begin{pmatrix} 1 & 0 & 1 \\ 0 & 1 & 1 \\ 0 & 0 & 1 \end{pmatrix}$	$\begin{pmatrix} 1 & 0 & 0 \\ 0 & 1 & 0 \\ \bar{1} & \bar{1} & 1 \end{pmatrix}$

Figure S3 Illustration of the transformation to the target lattice planes (hkl). All the directions are shown using stereographic projection of a cubic lattice vectors with the projection axis parallel to reciprocal lattice vector $\boldsymbol{T}^* = h_i \boldsymbol{a}_i^*$. Vectors $\boldsymbol{A}_1$ and $\boldsymbol{A}_2$ are parallel to the (hkl) planes and therefore fall on the great circle of the stereographic projection.

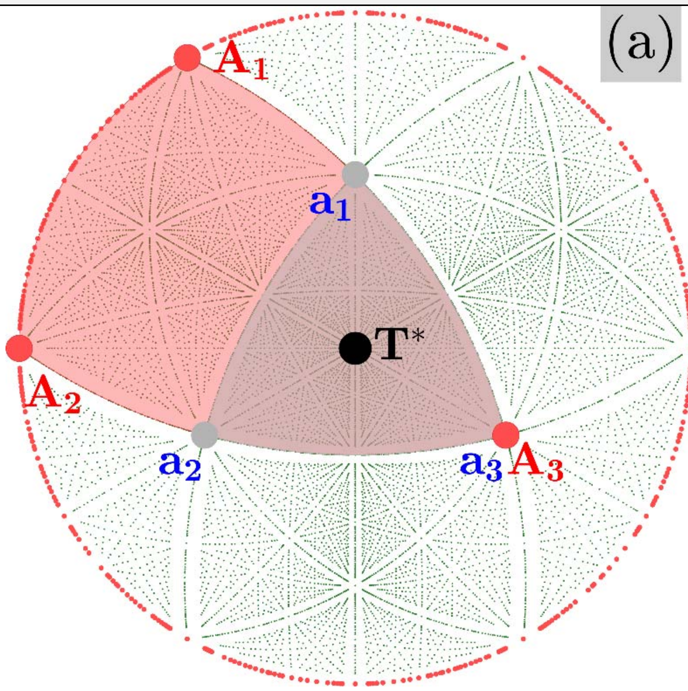

$(1\bar{2}3)$ $\begin{pmatrix} 0 & 1 & 1 \\ \bar{1} & \bar{2} & \bar{2} \\ 0 & 2 & 3 \end{pmatrix}$ $\begin{pmatrix} \bar{2} & 3 & \bar{2} \\ \bar{1} & 0 & 0 \\ 0 & \bar{1} & 1 \end{pmatrix}$

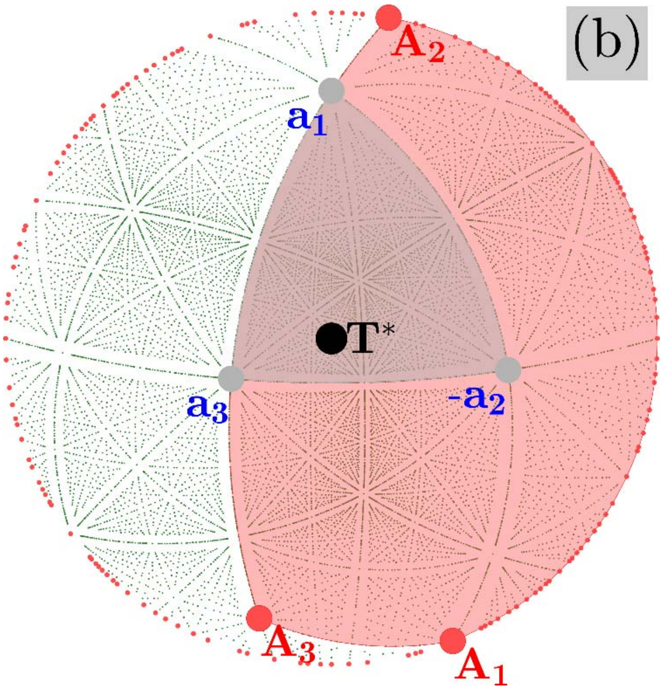

$(\bar{1}04)$ $\begin{pmatrix} 0 & \bar{1} & \bar{1} \\ 1 & 0 & 0 \\ 0 & 3 & 4 \end{pmatrix}$ $\begin{pmatrix} 0 & \bar{4} & 3 \\ 1 & 0 & 0 \\ 0 & \bar{1} & 1 \end{pmatrix}$

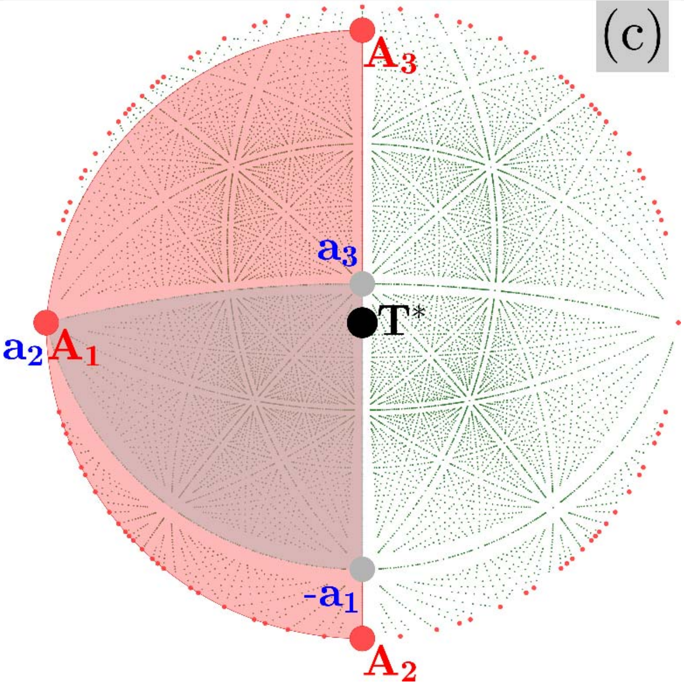

$$(335) \quad \begin{pmatrix} 1 & 2 & 3 \\ 0 & 2 & 3 \\ 0 & 3 & 5 \end{pmatrix} \quad \begin{pmatrix} 1 & 0 & 0 \\ \bar{1} & 5 & \bar{3} \\ 0 & \bar{3} & 2 \end{pmatrix}$$

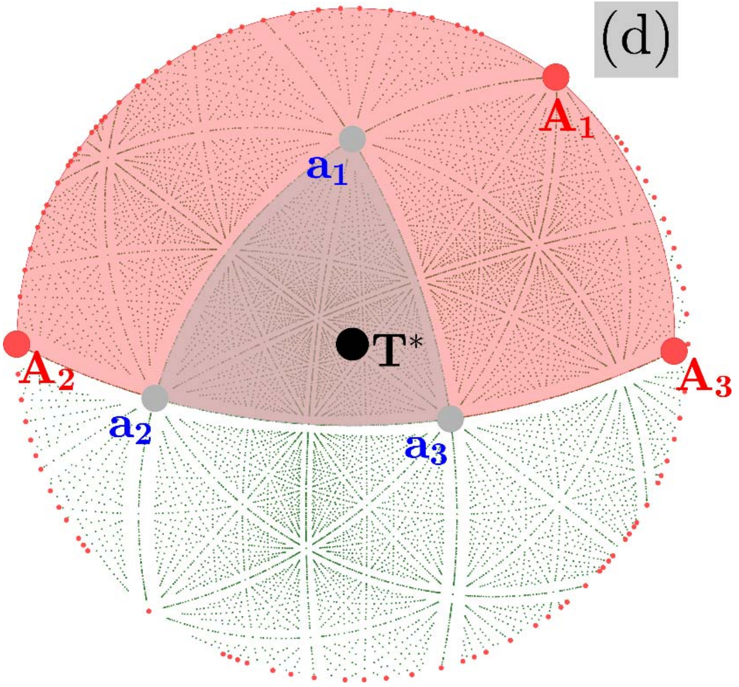

S5. Example of the application of the algorithm for the transformation of a unit cell of $LiNbO_3$ structure

The transformation of the crystal structure parameters (e.g. atomic coordinates, symmetry operations, atomic displacement parameters) is crucial for various crystallographic tasks. The analysis of a crystal growth, twinning, cleavage or inspection of two-dimensional structures of a surface may call for the change of the unit cell setting in such a way that one of the new basis planes (e.g. $\boldsymbol{A}_1\boldsymbol{A}_2$) becomes parallel to the specific planes (hkl). This transformation may e.g. help preparing a structure for surface and cleavage energy calculations, constructing supercells exposing a certain face of a crystal (Ong *et al.*, 2013; Tran *et al.*, 2016). The appropriate transformation of the basis vectors has already been described in the main section of the manuscript.

Here we will demonstrate this algorithm for the transformation of the unit cell of $LiNbO_3$. This structure has the space group type $R3c$ (Weis & Gaylord, 1985). The conventionally-chosen basis vectors form a non-primitive (R-centred) unit cell with "hexagonal" lattice parameters $a = 5.148, c = 13.863$ Å (Abrahams *et al.*, 1966). Although, the non-primitive unit cell is three times bigger than necessary, it simplifies the matrix-vector forms of the symmetry operations of the corresponding space group type (Hahn, 2005). Nonetheless, more economical description of the crystal structure requires a primitive unit cell, which is obtained by the transformation matrix $[S^{(p)}]$:

$$[S^{(p)}] = \begin{pmatrix} \frac{2}{3} & \frac{\bar{1}}{3} & \frac{\bar{1}}{3} \\ \frac{1}{3} & \frac{1}{3} & \frac{\bar{2}}{3} \\ \frac{1}{3} & \frac{1}{3} & \frac{1}{3} \end{pmatrix} \quad \text{(S5.1)}$$

The transformation to the primitive cell will change the original Miller indices of a plane as $[\boldsymbol{h}^{(p)}] = [\boldsymbol{h}][S^{(p)}]$. Then, the extension of the TRIO algorithm (§3) can be applied to obtain the target transformation of the primitive basis vectors to the lattice planes $(hkl)^{(p)}$. This transformation is expressed by the matrix $[S^{(hkl)^p}]$. According to (A7) the transformation of the original basis vectors are

$$[S^{(hkl)}] = [S^{(p)}][S^{(hkl)^p}] \quad \text{(S5.2)}$$

Table S4 and Figure S4 illustrate four examples of the transformation of the $LiNbO_3$ unit cell, along with the new lattice parameters $A_1, A_2, A_3,\ \alpha_1', \alpha_2', \alpha_3'$ calculated according to the equations (A6):

$$[G] = [S^{(hkl)}]^T [g][S^{(hkl)}], \quad \text{(S5.3)}$$

followed by

$$A_i = \sqrt{G_{(ii)}},\ \cos\alpha_i' = \frac{G_{(jk)}}{A_{(j)}A_{(k)}}\ (i, j, k \text{ are in cyclic permutation}) \quad \text{(S5.4)}$$

Such transformation can be used e.g. for simple and semi-empirical analysis of cleavage along different planes. The method of geometrical analysis of cleavage (based on the described transformation algorithms) will be reported in the upcoming publication in the same journal.

Table S4 Examples of transformation of the basis vectors to the target plane for $LiNbO_3$ structure. Column 1 shows the Miller indices of the target plane, relative to the original non-primitive (hkl) and primitive (S5.1) $(hkl)^p$ unit cell. Column 2 shows the transformation matrix (S5.2). Column 3 shows the lattice parameters of the new primitive unit cell whose basis vectors $\boldsymbol{A}_1$, $\boldsymbol{A}_2$ are parallel to the target lattice planes.

(hkl) $(hkl)^p$	$[S^{(hkl)}]$	A_1, A_2, A_3 $\alpha'_1, \alpha'_2, \alpha'_3$
(003) $(111)^p$	$\frac{1}{3}\begin{pmatrix} 3 & 0 & \bar{1} \\ 3 & 3 & \bar{2} \\ 0 & 0 & 1 \end{pmatrix}$	5.15 Å 5.15 Å 5.75 Å 116.56° 116.56° 60 °
(012) $(110)^p$	$\frac{1}{3}\begin{pmatrix} \bar{1} & 3 & \bar{1} \\ \bar{2} & 0 & 1 \\ 1 & 0 & 1 \end{pmatrix}$	5.76 Å 5.15 Å 5.76 Å 116.56° 53.13° 90°

Figure S4 The positions of Nb (green, larger circles) and O (brown, smaller circles) atoms in $LiNbO_3$ structure. The target lattice plane $\boldsymbol{A}_1\boldsymbol{A}_2$ is perpendicular to the plane of drawing and parallel to the horizontal direction. Such transformation / figures can be used to analyse e.g. cleavage properties, e.g. the existence of known cleavage plane (012) can be visually understood from Figure S5b.

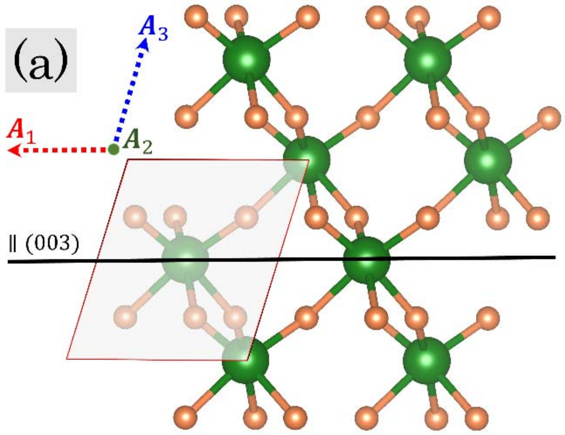

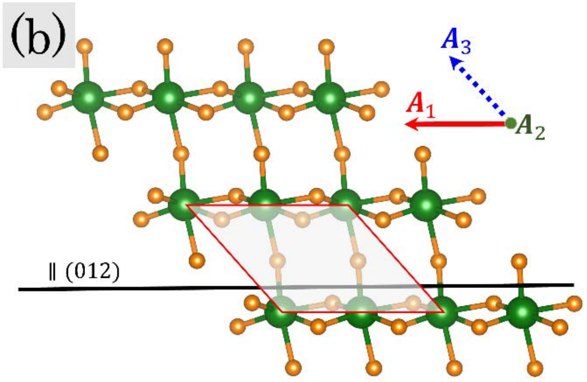

(110)<br>$(10\bar{1})^p$	$\frac{1}{3}\begin{pmatrix} \bar{1} & 1 & 1 \\ 1 & \bar{1} & 2 \\ 1 & 2 & \bar{1} \end{pmatrix}$	5.76 Å<br>10.30 Å<br>5.76 Å<br>153.44°<br>126.87°<br>47.87°	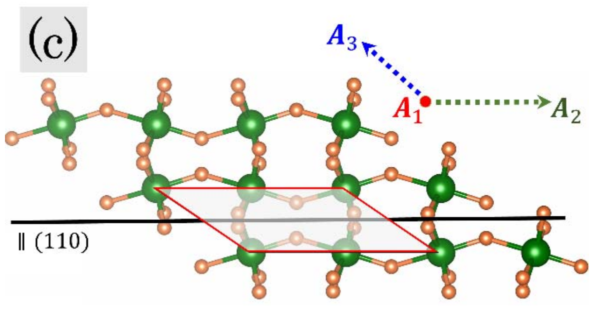
$(\bar{2}16)$<br>$(132)^p$	$\frac{1}{3}\begin{pmatrix} 5 & 3 & \bar{3} \\ 4 & 6 & \bar{3} \\ 1 & 0 & 0 \end{pmatrix}$	9.28 Å<br>8.91 Å<br>5.15 Å<br>150.00°<br>146.00°<br>50.17°	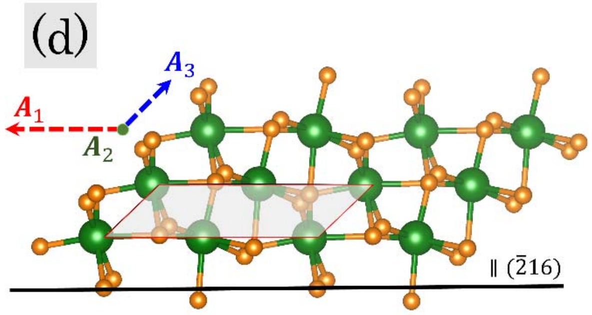
